# Specification of Self-Adaptive Privacy-Related Requirements within Cloud Computing Environments (CCE)

**DOI:** 10.3390/s24103227

**Published:** 2024-05-19

**Authors:** Angeliki Kitsiou, Maria Sideri, Michail Pantelelis, Stavros Simou, Aikaterini-Georgia Mavroeidi, Katerina Vgena, Eleni Tzortzaki, Christos Kalloniatis

**Affiliations:** PRIVASI Lab, Department of Cultural Technology & Communication, University of the Aegean, 81100 Mytilene, Greece; msid@aegean.gr (M.S.); mpantel@aegean.gr (M.P.); ssimou@aegean.gr (S.S.); kmav@aegean.gr (A.-G.M.); kvgena@aegean.gr (K.V.); etzortzaki@aegean.gr (E.T.); chkallon@aegean.gr (C.K.)

**Keywords:** self-adaptive, privacy, cloud computing environments, sociotechnical requirements, information disclosure, protection strategies, developer insights

## Abstract

This paper presents a novel approach to address the challenges of self-adaptive privacy in cloud computing environments (CCE). Under the Cloud-InSPiRe project, the aim is to provide an interdisciplinary framework and a beta-version tool for self-adaptive privacy design, effectively focusing on the integration of technical measures with social needs. To address that, a pilot taxonomy that aligns technical, infrastructural, and social requirements is proposed after two supplementary surveys that have been conducted, focusing on users’ privacy needs and developers’ perspectives on self-adaptive privacy. Through the integration of users’ social identity-based practices and developers’ insights, the taxonomy aims to provide clear guidance for developers, ensuring compliance with regulatory standards and fostering a user-centric approach to self-adaptive privacy design tailored to diverse user groups, ultimately enhancing satisfaction and confidence in cloud services.

## 1. Introduction

Cloud computing has ushered in a new era of technological innovation, offering unparalleled scalability, flexibility, and cost-efficiency to individuals and organizations. However, this rapid adoption of cloud services has also raised significant concerns regarding the privacy and security of sensitive data. In this section, we provide an overview of the evolution of cloud computing and outline the overarching privacy challenges that accompany its proliferation. The extensive collection, storage, and processing of data in cloud environments pose substantial risks to data privacy and security [1,2]. Traditional security measures may prove insufficient in addressing the sophisticated threats faced by cloud systems. The exploration of advanced encryption techniques, access controls, and data anonymization methods as potential solutions to safeguard data privacy in the cloud is needed. Vendor lock-in presents a formidable challenge for cloud users, limiting their ability to migrate between different providers and platforms. The analysis of the implications of vendor lock-in on service continuity, cost management, and innovation, and the discussion of strategies for mitigating lock-in risks through interoperability standards and open-source alternatives are immense tasks.

Compliance with data protection regulations is a paramount concern for cloud providers and users alike. Thus, the examination of the complexities of regulatory compliance in multi-jurisdictional cloud environments and the discussion of the role of transparent governance practices, privacy-enhancing technologies, and contractual agreements in ensuring compliance with relevant regulations are required. Transparency and accountability are foundational principles for building trust in cloud services. However, cloud users often lack visibility into the data handling practices of providers, leading to concerns regarding data sovereignty and user control. Therefore, the exploration of mechanisms for enhancing transparency and accountability, including clear data handling policies, user consent mechanisms, and audit trails, is also necessary. Multi-tenancy in cloud environments introduces challenges related to resource segregation and isolation, necessitating robust mechanisms to prevent unauthorized access and data leakage between tenants. Thus, the discussion of techniques such as hypervisor-based isolation, network segmentation, and containerization for enhancing resource segregation and ensuring tenant isolation in shared infrastructure environments is important. Cloud systems are vulnerable to malware attacks, data breaches, and insider threats, necessitating proactive measures for threat detection and mitigation. The analysis of the limitations of traditional intrusion detection systems and exploring emerging technologies such as machine learning-based anomaly detection and behavior analysis for real-time threat detection in dynamic cloud environments is also crucial [3,4].

Self-adaptive privacy plays a crucial role in addressing the privacy challenges inherent in cloud computing environments by providing dynamic and flexible mechanisms for protecting sensitive data and preserving user privacy. In particular, self-adaptive privacy mechanisms empower users to exert granular control over their personal data by allowing them to dynamically adjust privacy settings based on changing preferences, contexts, and risk levels. This level of control is essential in cloud environments where data may be shared across multiple applications and services, each of which has its own privacy requirements [5]. What is more, self-adaptive privacy solutions leverage contextual information such as user location, device type, and network conditions to dynamically adapt privacy protections in real time. By incorporating context awareness, these mechanisms can tailor privacy settings to specific usage scenarios, mitigating privacy risks associated with different contexts. Cloud computing environments are characterized by their dynamic and evolving nature, with new technologies, applications, and usage patterns constantly emerging. Self-adaptive privacy mechanisms are designed to adapt to these changes by continuously monitoring the environment and adjusting privacy controls accordingly, ensuring that privacy protection remains effective in the face of evolving threats and challenges [6,7].

Cloud computing environments are also susceptible to various privacy risks, including data breaches, unauthorized access, and insider threats. Self-adaptive privacy mechanisms employ risk assessment techniques to continuously evaluate the security posture of cloud systems and dynamically adjust privacy controls to mitigate emerging threats and vulnerabilities [8]. Compliance with data protection regulations such as GDPR, HIPAA, and CCPA is a critical requirement for cloud service providers and users. Self-adaptive privacy solutions enable automatic compliance monitoring and enforcement by dynamically adjusting privacy controls to ensure alignment with regulatory requirements and industry standards. By placing users at the center of the privacy decision-making process, self-adaptive privacy mechanisms empower individuals to make informed choices about how their data are collected, processed, and shared in the cloud. This user-centric approach enhances transparency, trust, and accountability in cloud environments, fostering a culture of privacy awareness and responsibility.

Overall, self-adaptive privacy is instrumental in addressing the complex and evolving privacy challenges in cloud computing environments by providing users with granular control, context awareness, risk mitigation capabilities, compliance assurance, user empowerment, and adaptability to change. By incorporating self-adaptive privacy mechanisms into cloud systems, organizations can enhance data privacy, security, and trust while enabling the responsible and ethical use of cloud services [9]. However, it has been identified that in self-adaptive approaches that have already been introduced, the social and technical privacy aspects that should be considered from both users’ and developers’ perspectives have been neglected. Our investigation has delineated three primary realms where self-adaptive privacy solutions find applications: Firstly, in algorithmic adaptiveness, particularly prominent in differential privacy algorithms manipulating relational datasets containing sensitive information. For instance, Li and Miklau [10] proposed mechanisms for differential privacy in counting queries, while Huo et al. [11] introduced real-time data aggregation for IoT with privacy considerations. However, these algorithms often overlook users’ social profiles and preferences, failing to address their privacy concerns adequately. Secondly, in software system adaptiveness, systems adjust their operations based on privacy policies or settings, yet frequently overlook crucial factors such as data collection limitations and minimization.

Díaz Ferreyra et al. [12] introduced PHeDer for encoding privacy practices, and Sanchez and Viejo [13] proposed a system reconciling privacy in web surfing and advertising. Wang and Srivastava [14] developed mPolicy for context-aware data-handling policies, and Kapitsak and Charalambous [15] proposed a privacy preference language for HTML5 web applications. Lastly, in user adaptiveness, where users define their privacy preferences, and information flow adjusts accordingly. Namara et al. [16], for instance, explored privacy adaptation methods for enhancing user engagement in social networks. Nevertheless, this approach sometimes disregards users’ contextual nuances and the technical hurdles they encounter in managing their information. This oversight erodes trust among users, systems, and providers, resulting in a dearth of privacy methodologies considered in cloud computing. In this regard and to address that, this paper presents the results of our previous mixed-methods research within the framework of our research project Cloud-InSPiRe.

The Cloud-InSPiRe project aims to push the boundaries of existing knowledge by proposing an interdisciplinary approach to enable the design of self-adaptive privacy-aware systems within cloud computing environments (CCE). This approach seeks to bridge the gap between social and technological aspects of privacy, recognizing the complex interplay between human behavior and technical infrastructure. A key innovation of the project lies in its expansion of the research area pertaining to self-adaptive privacy-related requirements within CCE. This expansion is crucial for capturing the socio-technical concepts essential for an integrated identification of privacy needs. Central to the project is the examination of privacy requirements under a unified framework. This framework will consider social, software, and infrastructure factors that are critical for the successful implementation of privacy measures. By highlighting the interdependencies among these factors, the project aims to develop a holistic approach to privacy design. Importantly, the project emphasizes the need to address the privacy needs of all stakeholders involved. This user-centric approach prioritizes the interests and concerns of individuals affected by privacy-related decisions, ensuring that the developed solutions are inclusive and effective. Ultimately, the project aims to deliver tangible outcomes in the form of an integrated framework and a beta-version tool for self-adaptive privacy design. These practical solutions will be informed by solid theoretical and methodological structures, drawing on insights from both sociological and privacy literature. In summary, the research motivation outlined underscores the project’s commitment to advancing knowledge and developing practical solutions for the complex challenges of self-adaptive privacy in CCE.

Under this light, two supplementary surveys have been elaborated [9,17], regarding (a) users’ needs for privacy within cloud computing environments based on their social groups and (b) developers’ perspectives on privacy, focusing on self-adaptive privacy in cloud environments. The integration of developers’ and users’ perspectives is crucial for confronting the challenges of self-adaptive privacy in cloud computing environments. The integration of these two perspectives—users’ social identity-based practices and developers’ insights and challenges—offers a comprehensive approach to addressing the complexity of privacy design in cloud systems. By aligning the design of self-adaptive privacy mechanisms with users’ social group affiliations and incorporating developers’ perspectives on privacy challenges and requirements, it becomes possible to develop more effective and user-centric privacy solutions, ensuring that privacy mechanisms are not only technically robust but also aligned with users’ needs and preferences, thereby enhancing trust and confidence in cloud services. Therefore, the contribution of this paper concerns the integration of technical, infrastructural and social requirements under a pilot taxonomy that can support and provide:

- A clearer guidance for developers in order to align technical measures with social needs effectively, helping them to understand the significance of social requirements.
- The empowerment of users with control over privacy settings, enhancing transparency and trust, proactively mitigating privacy risks, and granting users greater autonomy in privacy management.
- A user-centric approach that tailors privacy measures to diverse user groups, enhancing satisfaction.
- A compliant and standardized framework and best practices aiding in the improvement of self-adaptive privacy practices in cloud development.

The rest of the paper is structured as follows: Section 2 presents the findings of the aforementioned surveys focusing on social groups’ self-disclosure practices and self-protection strategies, thus highlighting users’ needs in the frame of privacy protection. Section 3 provides insights into the social, technical, and infrastructural requirements based on developers’ insights and users’ practices, while Section 4 provides a taxonomy for integrating self-adaptive privacy in cloud computing environments (CCE) according to these requirements. Finally, Section 5 discusses the importance of this approach that aligns social and technical requirements both in the frame of users’ needs to have more control over the information they upload while also mitigating risks and in the frame of developers’ work.

## 2. Background

**Figure 1:**
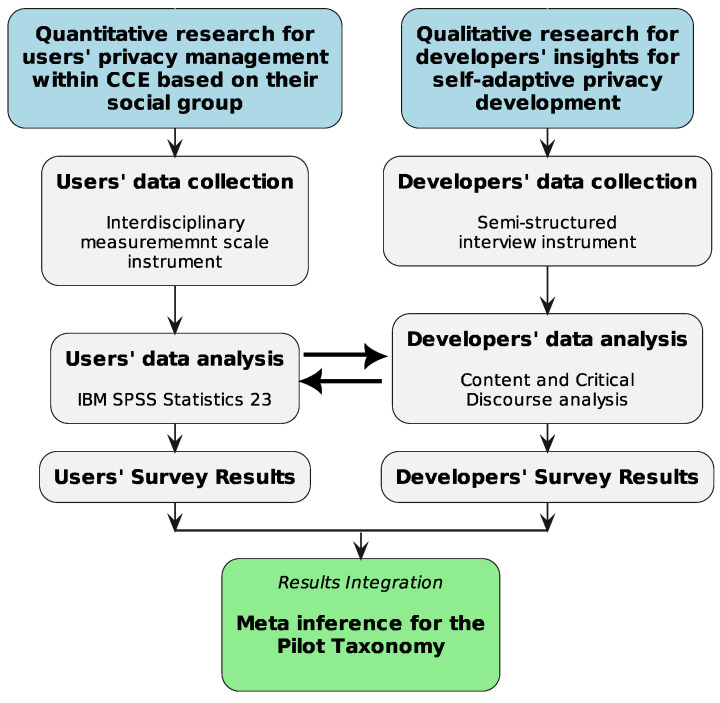
Cross-sectional empirical mixed-methods Research Design.

To offer insight into the identification of the complex factors that affect self-adaptive privacy-related Requirements, a cross-sectional empirical mixed-methods’ Research Design (RD) was elaborated. The interdisciplinary nature of the research prompts a reasonable balance among concepts from both the literature on sociology and privacy, addressing the following research questions: RQ 1: “Does belonging to a social group affect users’ privacy management within CCE?”, in order to understand users’ behavioral patterns, which will enable an optimal design for self-adaptive privacy-preserving schemes and RQ 2: “How do developers interpret the notion of privacy within CCE and how do they conceive the challenges for handling privacy requirements in a self-adaptive way?”, in order to meet efficiently both users’ social requirements and systems’ technical ones before performing adaptive privacy mechanisms. To address that, the RD included mixed quantitative and qualitative methods on the different research tasks (Figure 1). For RQ 1, the development of an interdisciplinary measurement scale, inbounding constructs, and validated metrics from both privacy and sociological literature was constructed and implemented in a research population, recruited from Greece, Spain, and the UK. The results were statistically analyzed via the “IBM SPSS Statistics 23” tool. For RQ 2, a semi-structured interview instrument was developed and applied to developers from Greece, Spain, and the UK, respectively, allowing them to demonstrate their presentations in depth. Content and critical discourse analysis were used for the results’ analysis.

The first supplementary survey focused on understanding users’ self-presentation and self-disclosure practices within cloud services, particularly in relation to their social group affiliations. By conducting a quantitative survey among students across universities in Greece, England, and Spain, the study provided valuable insights into how users’ social identities influenced their privacy behaviors [17]. In this regard, the foundational research question formulated to guide our study was RQ 1: “Does belonging to a social group affect users’ privacy management within CCE?”. These findings are significant for informing the design of self-adaptive privacy schemes tailored to different social groups’ needs. By incorporating users’ perspectives based on their social identities, developers can better understand the diverse privacy requirements of different user segments, thus improving the effectiveness of privacy mechanisms in cloud services.

The data collected suggested a diverse range of interests and motivations driving respondents’ participation in online groups, with a strong emphasis on social connection, professional networking, and recreational activities. The majority of respondents (33.9%) participated in companionship groups online. This indicated a strong desire for social interaction and connection in the online space, possibly for emotional support, friendship, or shared interests. Approximately 11.3% of respondents participated in professional groups online. This also suggested that a significant portion of respondents engaged in online networking for professional development activities related to their careers or fields of expertise. For the political, trade union, and voluntary groups, a smaller percentage of respondents participating in each group category was recorded (ranging from 2.4% to 8.1%). However, their presence indicated that some respondents were actively involved in civic engagement, activism, or volunteering efforts online. Around 11.7% of respondents participated in leisure groups, while 7.7% were involved in sport groups. A smaller proportion of respondents (5.9% and 2.9%, respectively) engaged in cultural and scientific groups online, indicating an interest in arts, culture, and intellectual pursuits within the digital sphere. Respondents also participated in groups focused on human support, environmental causes, gender equality, technology, religion, and mutual support, though each of these categories comprised a smaller percentage of participants. Some of the most important findings that can actually feed developers’ advances on self-adaptive approaches concerning (a) users’ self-presentation and information disclosure practices based on their social group and (b) users’ self-protection strategies are presented in Table 1, Table 2, Table 3, Table 4, Table 5 and Table 6.

The *chi-square* ($\chi^{2}$) statistic test was used to measure the differences between the observed frequencies of the outcomes of the set of our variables. $\chi^{2}$ was used to test whether two of the respective variables in each case were related or independent of each other. The different *p*-values indicate different types of hypothesis interpretations. p<0.05 (hypothesis interpretations are rejected); p≥0.05 (hypothesis interpretations are accepted). $\phi_{c}$ is also a *chi-square*-based measure of association. The *chi-square* coefficient depends on the strength of the relationship and sample size.

### 2.1. Groups and Self-Presentation and Information Disclosure

The results in Table 1 reveal the privacy behaviors of individuals across various social groups, shedding light on the nuanced approaches needed to safeguard personal information in online environments. Professionals, for instance, were cautioned when divulging job-related details and religious views, particularly on platforms like Messenger and Instagram, to maintain the delicate balance between privacy and professional boundaries. Similarly, members of trade union groups were urged to refrain from sharing photos and hobby-related information on platforms like Instagram, recognizing the potential implications for privacy. Furthermore, participants engaged in voluntary groups, spanning platforms like Instagram, were thoughtfully assessed for the information they disclosed regarding their own photos, to ensure robust privacy protection.

Likewise, individuals involved in cultural groups carefully managed the sharing of contact information, particularly on platforms like Instagram, to uphold their privacy rights. In this regard, the identification of privacy needs among different social groups is instrumental in informing the design, implementation, and optimization of self-adaptive privacy schemes. By tailoring privacy controls to match the preferences and behaviors of each group, these schemes empower users to make informed decisions about their online privacy. This tailored approach enhances user empowerment and fosters trust and transparency in online interactions. Additionally, leveraging contextual information allows for dynamic adjustments to privacy settings, ensuring they remain relevant and effective over time. Continuous adaptation and optimization based on user feedback further enhance the efficacy of these schemes, making them invaluable tools for promoting privacy awareness and user centricity in the digital landscape. The above confirms previous research regarding different social groups’ varying attitudes towards self-presentation and self-disclosure practices [18].

What is noteworthy, however (Table 2), is that these results differentiate when users belong to two groups simultaneously. Certain behaviors showed negative associations across different platforms. For instance, individuals participating both in companionship and professional groups were less likely to include contact information when using Messenger, as indicated by a statistically significant correlation ($\chi^{2}$ = 8.663, *p* = 0.003, $\phi_{c}$ = −0.245). Similarly, the negative correlations observed in sharing photos of oneself, sharing information about hobbies, and sharing information about daily activities on WhatsApp and Messenger platforms suggest for participants in both of the groups mentioned above a trend towards a more reserved behavior regarding self-disclosure in these contexts. For instance, individuals were less inclined to share photos of themselves on WhatsApp ($\chi^{2}$ = 12.038, *p* = 0.001, $\phi_{c}$ = −0.289) and information about hobbies on Messenger ($\chi^{2}$ = 8.456, *p* = 0.004, $\phi_{c}$ = −0.242). This cautious approach to sharing personal content may stem from concerns about privacy, security, or the potential consequences of sharing sensitive information in online environments, as previous research has highlighted [19].

Overall, these findings provide insights into how individuals navigate privacy boundaries in different social contexts and platforms, shedding light on the development of effective privacy protection strategies and the promotion of user trust and confidence in digital communication platforms.

### 2.2. Groups and Self-Protection Strategies

Furthermore, to also check if participation in a group was related to self-protection strategies, a chi-square test for two nominal variables was used. Results are shown in Table 5. As revealed, there was an association between the variables of participation in a companionship, professional, voluntary, leisure, scientific, environmental, religious, technological interest, and gender equality group and specific self-protection strategies. In all other cases of groups (political, trade union, sport, cultural, human support and mutual support), no statistically significant results were found (Table 3 and Table 4). The strength of association of the nominal-by-nominal relationships was positive in eight cases and negative in seven but low in all cases.

Once again, differences were noted when a user participated in two groups, as shown in Table 5.

The analysis of self-protection strategies within the context of companionship and professional aspects revealed noteworthy negative associations, indicating potential privacy vulnerabilities among participants. For instance, the reluctance to restrict access to uploaded content underscored the inadvertent exposure of personal or professional information to a broader audience. These findings accentuate the necessity for heightened privacy consciousness and proactive engagement in safeguarding personal and professional information within online environments, particularly concerning companionship and professional interactions.

Analyzing the self-protection strategies of individuals belonging to the “Companionship and Voluntary”groups, several notable patterns emerged. Indicatively, there was a negative association between disclosing personal information and group membership ($\chi^{2}\left(1\right)$ = 4.107, *p* = 0.043, $\phi_{c}$ = −0.170), supporting that individuals in these groups were more willing to share personal information. These findings underscored the nuanced privacy behaviors exhibited by individuals in the “Companionship and Voluntary”groups, emphasizing the importance of tailored privacy interventions and education within these social contexts.

Our second qualitative study discussed the evolving landscape of cloud computing services and the delicate balance between maximizing member interaction and safeguarding individual privacy. It highlighted the acknowledged need for self-adaptive privacy solutions that address both technical and social aspects in the cloud, particularly in light of increasing privacy requirements from providers and legislative frameworks like GDPR. The urgency for user-friendly privacy support solutions for developers was emphasized, as they play a crucial role in adhering to legal and technical privacy requirements and implementing user-centric privacy-friendly solutions. The survey findings indicated that privacy considerations often took a back seat in the adoption of cloud computing, revealing a tension between technological advancement and privacy protection.

Despite the growing importance of privacy and the frequency of data breaches, developers faced challenges in prioritizing privacy over technical and business factors. The lack of focus on “self-adaptive privacy”among developers suggested a gap in user-centric privacy features, highlighting the need for more nuanced and adaptive privacy controls aligned with users’ preferences. Developers expressed a desire for tools and frameworks to seamlessly integrate privacy into cloud services, underscoring the industry’s need for standardization. Standardized methodologies and frameworks can provide a structured approach to self-adaptive privacy integration, reducing inconsistencies and oversights.

Overall, addressing the multifaceted landscape of privacy in cloud computing requires a holistic approach that considers infrastructure selection, development methodologies, user-centric controls, transparency, and ongoing compliance. Achieving a balance between privacy and business priorities emerges as a central challenge, necessitating collaborative efforts, education, and the development of practical tools and frameworks. The proposed taxonomy of self-adaptive privacy-related requirements offers a practical step for developers and organizations to navigate these challenges effectively, clarifying responsibilities and enhancing engagement to improve privacy practices.

## 3. Social, Technical, and Infrastructural Requirements Based on Developers’ Insights and Users’ Needs

This section provides the self-adaptive privacy-related requirements based on the findings from developers’ perspectives and social identity-based user practices.

### 3.1. Social Requirements

These social requirements underscore the importance of incorporating self-adaptive privacy-enhancing features into cloud platforms to address the specific privacy concerns and preferences of users engaged in several activities. Below are the findings of social requirements by user group for self-presentation and information disclosure.

#### 3.1.1. Groups and Self-Presentation and Information Disclosure

*Users belonging to the professional group*

- Social requirement 1: platforms should provide robust privacy settings, allowing users to manage the visibility of their religious views, especially on Messenger.
- Social requirement 2: users should have the option to include or exclude a short curriculum vitae (CV) in their profile, with clear controls over its visibility to different audiences, particularly on Instagram.
- Social requirement 3: platforms should offer users granular control over photo-tagging permissions to manage the visibility of tagged photos, especially on platforms like Instagram, where users frequently tag others in shared photos ($\chi^{2}\left(1\right)$ = 5.549, *p* = 0.018, $\phi_{c}$ = −0.142).

*Users belonging to the trade union group*

- Social requirement 1: users engaging in trade union activities show a preference for privacy regarding sharing photos of themselves on Instagram, indicating a need for platforms to implement robust privacy controls for image sharing functionalities.
- Social requirement 2: Users involved in trade union activities are more cautious about sharing information about their hobbies, especially on platforms like Instagram. This underlines the importance of providing users with granular control over the visibility of hobby-related information on their profiles.
- Social requirement 3: Trade union members are concerned about sharing their location on social media, particularly on Instagram. This highlights the necessity for platforms to offer effective location privacy settings and raise awareness among users about the risks associated with sharing their whereabouts.
- Social requirement 4: Users affiliated with trade unions tend to avoid tagging others in photos they share on Instagram, indicating a desire for stricter control over the dissemination of personal information. Platforms should enhance tagging mechanisms to ensure users have the autonomy to manage tags and protect the privacy of themselves and others.

*Users belonging to the voluntary group*

- Social requirement 1: Users engaged in voluntary activities exhibit a preference for privacy regarding sharing photos of themselves, particularly on Instagram. This indicates a need for social media platforms to implement robust privacy controls and provide users with options to manage the visibility of their photos, ensuring that individuals involved in voluntary work can maintain their privacy while using the platform.

*Users belonging to the cultural group*

- Social requirement 1: Users associated with cultural groups demonstrate a reluctance to share contact information, particularly on Instagram. This highlights the importance of respecting users’ privacy preferences regarding the disclosure of contact details within the context of cultural activities.

*Users belonging to the scientific group*

- Social requirement 1: Users affiliated with scientific groups exhibit a tendency not to share information about their hobbies on Instagram. This underscores the necessity for privacy safeguards concerning the disclosure of personal interests within scientific circles on social media platforms, specifically Instagram.
- Social requirement 2: Users involved in scientific groups refuse to share information about their daily activities on Messenger. This underscores the significance of privacy protection mechanisms concerning the disclosure of routine behaviors within scientific communities on messaging platforms like Messenger.

*Users belonging to the environmental group*

- Social requirement 1: Users engaged in environmental groups demonstrate a tendency not to share personal information on Messenger. This highlights the importance of implementing privacy measures to safeguard personal data exchanged within environmental communities on messaging platforms like Messenger.

*Users belonging to the technological interest group*

- Social requirement 1: Users affiliated with technological interest groups demonstrate a tendency not to share photos of themselves on Instagram. This underscores the importance of implementing privacy controls and mechanisms on Instagram to empower users to manage their photo sharing preferences effectively.
- Social requirement 2: Users affiliated with technological interest groups demonstrate a tendency not to share information about their hobbies on Instagram. This emphasizes the necessity of enhancing privacy features that enable users to selectively share hobby-related content with specific audiences.
- Social requirement 3: Users engaged in technological interest groups manifest a tendency not to share information about their daily activities on Instagram. This highlights the need to enhance privacy settings and allow users to regulate the visibility of their daily activity updates.

*Users belonging simultaneously to the companionship and professional groups*

- Social requirements for users of WhatsApp: there is a need for robust privacy controls and mechanisms for sharing personal information, photos, information about hobbies, and daily activities on this platform to safeguard their privacy.
- Social requirements for users of Facebook: there is a need to freely share information about their friends.
- Social requirements for users of Messenger: there is a need for privacy controls when sharing information regarding daily activities, sexuality, religious views, contact information, or location.

#### 3.1.2. Groups and Self-Protection Strategies

As far as the self-protection strategies concern, we identified the following requirements per group:

*Users belonging to the companionship group*

- Social requirement 1: individuals in companionship groups should frequently adjust their privacy settings to maintain control over their shared content.

*Users belonging to the professional group*

- Social requirement 1: professionals should refrain from not restricting access to the content they upload to ensure privacy and professional boundaries.

*Users belonging to the voluntary group*

- Social requirement 1: participants in voluntary groups are advised to untag themselves from others’ photos to manage their privacy effectively.

*Users belonging to the scientific group*

- Social requirement 1: individuals in scientific groups should consider changing their initial privacy settings in their profiles to mitigate privacy risks.
- Social requirement 2: individuals in scientific groups should consider using a limited profile option to control their visibility.
- Social requirement 3: individuals in scientific groups should consider excluding contact information from their profiles to mitigate privacy risks.

*Users belonging to the gender equality group*

- Social requirement 1: participants in gender equality groups should be cautious not to accept friendship requests from strangers to mitigate privacy risks

*Users belonging simultaneously to the companionship and professional groups*

- Social requirement 1: platforms should offer the option for pseudonymous or anonymous registration to accommodate users’ preferences for privacy, especially for users who choose not to provide their real names during registration.
- Social requirement 2: Platforms must enhance their privacy settings to allow users who participate in both groups to have greater control over who can access the content they upload. This includes implementing robust access control mechanisms to safeguard users’ privacy preferences.

*Users belonging simultaneously to the companionship and voluntary groups*

- Social requirement 1: platforms should introduce features that enable users to make informed decisions regarding personal information disclosure, including information about their families, to safeguard users’ privacy preferences.

### 3.2. Technical Requirements

Technical requirements were extracted during previous research (Kitsiou et al. [9]), after discussing them with developers during a free-form interview. The second research question that had to be addressed was RQ 2: “How do developers interpret the notion of privacy within CCE and how do they conceive the challenges for handling privacy requirements in a self-adaptive way?”. A further analysis revealed seven major technical and three infrastructural requirements to consider for implementing a self-adaptive privacy enabled application. Technical requirements mostly boiled down to controls and available tools users and developers needed, while infrastructural requirements referred to cloud infrastructure and methods mostly used during development.

*TR-1 Compliance management:*

Compliance management integrates all the legal and regulatory compliance, along with robust verification mechanisms and service-level agreements (SLAs). It is a critical requirement for designing self-adaptive privacy systems, and it should be considered at the early stages of the system’s design/development process. Integrating privacy-by-design principles into the development process makes privacy aspects part of the solution from the outset and throughout the complete development process. In the context of self-adaptive privacy, these systems need to adhere to various laws and regulations to ensure that users’ personal data and information are handled appropriately. The emphasis on compliance mechanisms provided by cloud providers suggests an approach to meeting regulatory requirements [5]. The system should be designed in a flexible form to adapt to changes in privacy laws and regulations. This adaptability ensures continued compliance as legal frameworks evolve.

Due to multiple jurisdictions, there are different data protection laws, such as the General Data Protection Regulation (GDPR) or the California Consumer Privacy Act (CCPA), that impose strict requirements on how personal data should be handled. Designing self-adaptive privacy systems involves ensuring compliance with these laws to avoid legal penalties and maintain user trust. Implementing compliance verification mechanisms within the privacy system is essential. This involves regularly auditing and assessing the system’s adherence to legal and regulatory requirements.

Compliance mechanisms provided by big cloud providers suggest a pragmatic approach to meeting regulatory requirements. The desire for tools that facilitate auditing and verification indicates a recognition of the importance of validating privacy measures. The previous statement is mentioned by the developers, and it aligns with the broader trend in software development towards continuous testing and validation. Additionally, the suggestion to avoid manual compliance checks highlights the desire for automation in the compliance process. Manual checks can be time-consuming and prone to errors, and developers are looking for ways to streamline this aspect of their workflow.

Robust SLAs should be implemented and signed by all parties to understand all the legal and technical aspects of rights and obligations. There should be guidance in the selection process of the appropriate SLA regarding privacy, and the SLAs should be customized to include the users’ needs. The SLA terms should be clear, ensuring accountability along with reliability and also ensuring that self-adaptive privacy systems meet the necessary standards and mitigate the risk of non-compliance penalties or lawsuits. SLAs can include provisions for continuous improvement and adaptation of privacy measures in response to changing regulatory requirements, technological advancements, or emerging privacy risks.

*TR-2 Transparency awareness:*

Establishing transparency awareness is crucial. It ensures that consumers have a clear understanding of the control and management of their own data. Consumers should know at all times how their data are handled, where they reside, what security and privacy measures are taken, and who can access them. Users should be provided with clear and understandable information about the management of their data, allowing them to make informed decisions and provide meaningful consent based on their social group norms. Taking away this requirement, consumers cannot self-adapt their private needs since they depend on providers’ good will to fulfill their demands. In many cases, cloud providers offer users limited privacy choices, often leading to users’ lack of visibility. Additionally, providers can enable data collection mechanisms to gain access to users’ data without users being aware of these activities.

Transparent communication about data practices is crucial for building users’ trust. The system should collect only the necessary data for a specific purpose and ensure these data are not used beyond the intended scope without the user’s explicit consent. Consequently, it should incorporate mechanisms for obtaining this explicit consent for data processing activities. Transparency also helps mitigate privacy risks by enabling early detection and response to potential privacy issues. When consumers are informed about data breaches or any other incident, they can act accordingly. They can change their passwords or adjust their privacy settings to meet their needs.

A further complicating matter is the diverse legal jurisdiction in countries where cloud services are hosted, resulting in varied definitions of privacy protection and multiple frameworks for its applicability. The implementation of different privacy controls or mechanisms to comply with specific regulations should be built in a transparent manner and communicated to consumers. Additionally, service level agreements (SLAs) contribute to transparency by providing users with clear and understandable information about the privacy practices and commitments of the service provider. This transparency helps build trust and confidence among users, as they know what to expect regarding the handling of their personal data.

*TR-3 Context awareness:*

Designing self-adaptive privacy systems that effectively balance privacy protection with user preferences and contextual factors necessitates a comprehensive understanding of context awareness [20]. The ability of systems to record and analyze various users’ characteristics, such as user activity, location, and social interactions, so that they are able to make informed decisions about privacy settings, is crucial [21]. By collecting these data, systems can gain valuable insights into the specific circumstances of data usage, enabling the customization of privacy settings that are tailored to the individual needs and preferences of each user. Furthermore, verifying the satisfaction of users’ privacy objectives in software design involves assessing how users’ awareness, or lack thereof, regarding the disclosure of information relates to the communication properties of objects within the design.

Privacy is acknowledged as a context-dependent concept, varying according to the context, situation, and perspective of the individual and thus privacy-related values have been found to be expressed in multiple and conflicting ways [22]. In summary, context awareness is critical for developing self-adaptive privacy protection systems that successfully balance user preferences, contextual considerations, and privacy protection.

*TR-4 User interaction:*

User interaction in systems is crucial for empowering users to understand, manage, and provide feedback on their privacy settings effectively. Additionally, social interaction and feedback are important for designing and implementing more effective systems, supporting developers’ aims [23]. Also, education empowers users to recognize potential threats and their role in safeguarding their privacy within self-adaptive systems. By understanding how these systems operate, users can decide about their privacy settings, appreciate their value, and engage more actively in managing them [24]. Moreover, providing mechanisms for users to offer feedback on their privacy preferences enables a continuous improvement and adaptation of these systems. Additionally, notification management plays a crucial role in user interaction. Fine-grained notification controls are essential for preserving user privacy while maintaining a positive user experience.

In summary, user interaction requirements in self-adaptive privacy systems encompasses educating users about privacy implications, facilitating feedback mechanisms for refining privacy settings, involving users in the design process, and enhancing notification management for a seamless user experience. Prioritizing user engagement and feedback is a key to building trust, improving system effectiveness, and ultimately safeguarding user privacy in an increasingly complex digital landscape.

*TR-5 PII attribute management:*

Personally Identifiable Information (PII) attribute management is essential when applying self-adaptive privacy techniques. The identification of PII attributes and their categorization help developers and users decide the actions and measures to fine-tune privacy controls according to their needs. During the previous stage of our research, we identified different kinds of attributes, such as (a) identity attributes (names, identity numbers, usernames, emails, etc.), (b) social attributes (professional, beliefs, religion, political opinions, sexual orientation, social interactions, etc.), and (c) location attributes (addresses, GPS traces, routes, etc.). All these attributes must be explicitly identified during the development phase so that the appropriate handling methods are available to the end user after application deployment. Each attribute should have a set of “operations”available regarding self-adaptive privacy so that end users can set their preferences. Such operations include but are not limited to:

- The protection of an attribute from unauthorized access;
- The minimization of an attribute when it is not required for some operation;
- The retention of an attribute according to user preferences and applicable laws;
- Anonymization when required;
- Encryption when required.

Organizing PII attributes this way and specifying available operations will provide the developers with a systematic way to act on sensitive information while organizing their development procedures against these operations. On the other hand, end users will also benefit because setting their PII preferences on their part will be easier and could be guided. Such a formalization will also enhance interoperability between different systems because the definitions of operations could be independent of the underlying infrastructure.

*TR-6 Privacy control:*

When developing self-adaptive privacy applications, several technical requirements must be considered, particularly in the domain of privacy control. Firstly, algorithmic adaptiveness is crucial, especially for differential privacy algorithms dealing with relational datasets containing sensitive information. Secondly, software systems must exhibit adaptiveness to varying privacy policies and user settings, ensuring compliance with data collection limitations and minimization principles. Thirdly, privacy management should empower users to choose their desired level of personal information protection, acknowledging their strategic disclosure behaviors for online identity projection. Additionally, by incorporating adaptive privacy management systems, encryption mechanisms, and role-based access controls, the management of privacy preferences, concerns, and rights is facilitated. User adaptiveness is essential, allowing a dynamic adjustment of privacy preferences to regulate information flow accordingly. Furthermore, enabling user-driven privacy control through dynamic updates of consent settings and versioning of privacy policies ensures transparency and user empowerment. Finally, effective user content management mechanisms should be implemented to further enhance privacy control capabilities.

*TR-7 Intelligence management:*

Intelligence management within self-adaptive privacy applications is crucial for understanding privacy requirements as fundamental quality attributes and embedding them as constraints throughout the development process to ensure end-user satisfaction. Integrating machine learning and artificial intelligence mechanisms is essential for comprehending user preferences, behaviors, and privacy needs. These mechanisms enable the development of adaptive privacy policy prediction systems, tailored for scenarios such as user-uploaded images on content sharing sites, and recommendation systems based on social behavior to enhance privacy protection, particularly concerning sensitive life aspects. However, challenges arise in real-time data analysis, transparency, and resource allocation in cloud data centers, necessitating efficient solutions for resource segregation and access to substantial volumes of big data. Additionally, adopting machine-readable privacy specification languages streamlines the expression and enforcement of context-aware privacy policies, offering a standardized framework for by-product data management in privacy-sensitive contexts.

### 3.3. Infrastructural Requirements

*IR-1 Storage management:*

Storage management plays a crucial role in designing self-adaptive privacy systems, which aim to dynamically adjust privacy controls based on changing requirements, user preferences, and contextual factors. The Cloud Storage Guidelines are a comprehensive collection of recommendations designed to ensure the safe and respectful use of data in cloud computing environments. Data management in a secure storage environment entails several critical principles, such as data classification, which entails the categorization of data based on their sensitivity, encryption policies, and access controls. Furthermore, specific criteria for the full data lifespan, which includes creation, archiving, retrieval, and deletion, are required. This technique places a strong emphasis on adhering to retention schedules.

The storage location is also an important consideration since it includes the geographic locations of data centers and ensures compliance with local data protection legislation, particularly in circumstances where data sovereignty is a major concern. Additionally, data integrity tests are critical for ensuring correct and dependable data, along with data redundancy and regular backups, which reduce the risk of data loss while also maintaining data availability and recoverability in the case of system failure or corrupted data. It is also critical to evaluate data portability possibilities, particularly when considering prospective cloud provider transfers. This includes considering how to convey data without jeopardizing security or privacy.

Another aspect of data management is vendor lock-in mitigation strategies. These strategies are key considerations for tackling the potential risks associated with being overly reliant on a single cloud service provider. Using containerization and orchestration solutions such as Docker and Kubernetes, portability is improved. These solutions wrap applications and their dependencies in containers, allowing for easy portability across several cloud environments. Simultaneously, while engaging in contracts with cloud service providers, organizations should consider flexibility and data retrieval options. They should also include clear exit clauses and well-defined procedures for moving to a new supplier in contractual agreements to ensure adaptability. Regular interoperability testing is critical for identifying and addressing any difficulties in the movement of apps and data between cloud providers before a full-scale relocation is required. Prioritizing data portability and proper planning entails determining how readily data and apps can be transferred between cloud providers with minimal disturbance, with a focus on using standardized formats to ensure smooth transitions. Finally, implementing a multi-cloud strategy, which entails dispersing workloads over multiple cloud providers, ensures redundancy, resilience, and the ability to select services based on each provider’s strengths.

*IR-2 Collaboration:*

Collaboration has a significant impact on users’ privacy experiences. Users with similar social identities, frequently based on friendship or shared interests, build collaborative communities in which members feel more comfortable providing personal information, thus increasing trust. The implementation of a Role-Based Access Control (RBAC) model emphasizes a collaborative approach to privacy management. Unlike typical rule-based paradigms, RBAC considers users’ social roles, including the implicit privacy preferences that come with each role. The use of a hybrid mining method exhibits collaboration between data mining techniques and social context comprehension, resulting in meaningful social roles that capture users’ privacy and interests collectively. Incorporating users’ feedback at each stage of the privacy settings recommendation process improves collaboration by allowing users to actively participate in the refinement of settings based on their experience and preferences. This collaborative feedback loop ensures that privacy measures are more aligned with user expectations. Furthermore, acknowledging the delicate balance between users’ desires for social capital and the need to protect privacy represents a collective effort to develop systems that allow users to effectively negotiate this equilibrium. Overall, cooperation is built into the fabric of social platform privacy, ensuring that measures are not imposed unilaterally but rather cooperatively designed with users’ feedback and context in mind.

*IR-3 Developer Guidance:*

Developer guidance aims to help developers cope with the requirements of self-adaptive privacy during application development. We identified the need for action in two directions, namely, tools and practice. First, tools must provide developers with easy-to-use functionality to document and analyze the application under development. Such documentation tools should not only track the state of privacy handling during the development stage but also help to construct a deliverable that will enhance transparency at a later stage. Moreover, this kind of documentation tool will help to bridge the gap between privacy experts/legal departments, providing a means of collaboration and discussion. Analysis tools will help with the identification of PII information relative to an application and guide developers toward the appropriate handling according to the TR-5-PAM requirements. This could also help developers prioritize data-handling operations according to their importance or impact. Regarding developers’ practices, we propose extra steps in the developers’ methodology to consider during development [25]. These steps should be a “discussion”and “action-needed”phase injected into whichever methodology is used at the appropriate stage. For example, after discussing a new feature and setting the requirements, there should be a privacy-related discussion about that feature afterwards regarding privacy impacts. These steps should also include legal/privacy experts. This sort of formalization will help to change developers’ mentality regarding privacy and standardize the process, while it could be assisted by the documentation tool, as discussed earlier.

## 4. A Taxonomy for Integrating Self-Adaptive Privacy Requirements Informed by Both Developers’ Insights and Social Identity Considerations

To align technical and social requirements, we further encoded social requirements and social protection measures by grouping them by similarity among different users’ groups. We identified 7 distinct social requirements, SR-1 to SR-7, and 10 distinct social protection measures, SP-1 to SP-10. Social requirements can be found in Table 7.

Social protection measures can be found in Table 8. We can further merge social protection measures as extracted from our research with social requirements, since those might overlap regarding their actual impact. That is, SP-1, SP-2, and SP-9 are included or can be merged in SR-1, SP-3 in SR-3, and SP-6 in SR-6. Furthermore, we introduce new requirements as follows: SP-4 becomes SR-8, SP-5 and SP-8 become SR-10, SP-7 is SR-11, and SP-10 is SR-9. Note that the order of the numbering is not representative of the requirements’ significance or importance. The final list of the identified requirements is presented in Table 9.

Regarding users groups, the above social requirements are mapped in Table 10.

As we can see, the professional and companionship groups have more requirements than the rest of the groups, followed by the scientific group. Having this encoding applied, we can align social and technical requirements in a way that it is obvious which requirement is supported by which, thus allowing us to further focus on the implementation details for each requirement. This alignment is presented in Table 11.

For each social requirement (SR), its alignment with technical requirements (TR) and infrastructural requirements (IR) is explained below.

As far as SR-1 is concerned, users should be informed about the context in which their information is shared (TR-3). User feedback and notifications (TR4) are important when managing visibility. Users should also be able to manage their PII information (TR-5) and select the type of protection they need (TR-6). Systems intelligence could also guide users (TR-7) to take informed actions. Finally, collaboration tools are essential when dealing with different groups and need to adjust privacy settings (IR-2).

Regarding SR-2, when dealing with this option, users should be well informed about the context in which information will be shared (TR-3) and have the required controls to alter this option (TR-6).

For requirements SR-3, SR-4, SR-5, and SR-7, users should be aware of their context when sharing information (TR-3). The system should be able to notify the user when photos are tagged to take the required actions or set related notifications (TR-4). Controls for such actions should be available to the users (TR-6). System intelligence could also assist users in taking the required actions as needed (TR-7).

Regarding SR-6, it is important to adhere to compliance requirements (TR-1), while users are well informed about the way each provider handles sensitive information (TR-2). Control of personal data disclosure can be managed through the appropriate controls (TR-5). For this requirement, storage management is also important, and the users must be informed and apply their preferences (IR-1).

Regarding SR-8 and mitigating privacy risks, it is important for users to understand how the system handles their information (TR-2 and IR-1) and take the required steps through the available settings (TR-5 and TR-6). The system’s intelligence could also assist users in making better choices according to their needs (TR-7).

Regarding SR-9, it is important for end users to be informed about compliance requirements (TR-1). Transparency and context awareness are also essential for appropriate user decisions (TR-2, TR-3). User interaction with the system regarding feedback and notifications (TR-4) assists users in taking appropriate actions through the available controls (TR-6). Intelligence management could offer more insight to users and their actions (TR-7). This is obviously the most intense requirement, which relates with most of the technical requirements.

For SR-10, it is important for the users to understand how their information will be handled (TR-2) and provide the required obfuscated information (TR-5)

Finally, to achieve SR-11, users must be aware of the context where requests come from (TR-3) and adjust their settings (TR-6). The system’s intelligence could assist users in making better choices according to their needs (TR-7). Collaboration mechanisms and management are also essential for association management (IR-2)

As we can see, the most demanding are SR-1 and SR-9, which span most of the technical and infrastructural requirements. On the other axis, TR-2, TR-4, and TR-6 seem to have more significance than social requirements. Developer guidance (IR-3) is relevant to all social requirements, since developers should be aware of each one separately. Moreover, they should be able to apply all required technical measures available to ensure user privacy is protected appropriately according to their choices.

## 5. Discussion and Conclusions

The integrated approach, as described, focuses on aligning social requirements with technical ones to enhance self-adaptive privacy practices. By encoding social requirements and mapping them to technical requirements, developers can ensure that cloud development aligns with users’ privacy needs, thus confronting problems and needs that previous research has acknowledged [26,27]. The integrated approach facilitates the implementation of robust privacy settings (TR-1) on cloud platforms, allowing users to manage the visibility of their content among different groups (SR-1) [26]. This helps in complying with regulations and addressing privacy concerns effectively. TR-1 emphasizes the importance of adhering to compliance requirements, ensuring that platforms handle sensitive information appropriately, aligning with the privacy preferences outlined in SR-6. TR-2 ensures that users are well informed about how providers handle their sensitive information, enhancing transparency and trust, which is also essential for SR-6. What is more, TR-2 and TR-5 help users understand how the system handles their information and enable them to take required steps to mitigate privacy risks, aligning with SR-8 and SR-9. TR-3 ensures that users are well-informed about the context of information sharing, which is essential when considering whether to include or exclude a CV in their profile (SR-2), while it also ensures that users are aware of the context when sharing information, aligning with the granular control requirements of SR-3, SR-4, SR-5, and SR-7. TR-4 emphasizes the significance of user feedback and notifications in managing visibility, which complements SR-1 by empowering users to control the visibility of their content effectively, while it notifies users about photo-tagging activities, enabling them to take the required actions to protect their privacy, which is essential for SR-3 and SR-7.

Overall, the integrated approach enables developers to implement comprehensive privacy practices in cloud development by addressing both technical and social requirements, thereby enhancing user trust and compliance with privacy regulations. TR-5 and TR-6 enable users to manage their PII and select the type of protection they need, aligning with the granular control requirements outlined in SR-1. TR-6 provides users with the required controls to alter this option, empowering them to make informed decisions about their profile visibility, while it helps users manage the visibility of tagged photos and other personal information, supporting the granular control requirements outlined in SR-3 and SR-4. Finally, TR-7 plays a crucial role in guiding users to take better actions, ensuring that privacy settings align with their preferences and requirements, while it assists users in making better choices according to their needs, enhancing their ability to make informed decisions, which is crucial for SR-9 and SR-11. The alignment of technical requirements with social requirements contributes to improving privacy practices in cloud development by empowering users with greater control over their privacy settings and enhancing transparency and trust in information sharing [7].

Developers play a crucial role in implementing these measures effectively and ensuring that users’ privacy is protected appropriately according to their choices and preferences. In this regard, clear guidance should be provided to developers on how to align technical measures with social requirements effectively, especially since previous research has underlined the gap in understanding the interpretation of privacy, requests, and challenges faced by developers [28]. This could include standardized frameworks and best practices for implementing privacy controls in cloud applications. Developers should be educated on the significance of each social requirement and its corresponding technical measure, ensuring that they understand the rationale behind privacy protection measures.

Actionable insights can be derived from user feedback and system intelligence to improve the effectiveness of privacy controls and address emerging privacy concerns. Training programs and resources should be made available to developers to enhance their skills in implementing self-adaptive privacy measures and social identity-based privacy schemes. Therefore, the suggested taxonomy, based on the alignment of social and technical requirements in cloud development, signifies a pivotal shift towards enhanced user empowerment and improved privacy protection. By synchronizing technical measures with specific social needs, users are granted greater autonomy in managing their privacy preferences across various platforms and user groups.

This alignment not only fosters trust and transparency between users and service providers but also proactively mitigates privacy risks by implementing targeted technical solutions. It ensures that users remain confident in sharing their information, knowing that robust privacy measures are in place to safeguard their data and adhere to compliance standards. Moreover, this alignment streamlines the development process, providing developers with clear guidance on integrating privacy features effectively.

By adopting a user-centric approach, platforms can tailor privacy measures to meet the diverse needs of different user groups, ultimately enhancing overall user satisfaction [4]. This cohesive integration of social and technical requirements underscores a commitment to prioritizing user privacy, fostering a privacy-first mindset among developers and service providers, and ensuring compliance with regulatory standards.

Furthermore, self-adaptive privacy mechanisms can enhance the privacy of users’ data shared within social sensing frameworks. Nunes et al. in [29] already discussed the potential of people-centric sensing systems, which integrate mobile phones and sensors with social networks like Facebook, to expand social networking usage, thus highlighting challenges such as privacy concerns and heterogeneous sensor networks. By dynamically adjusting privacy settings based on user preferences, context, and privacy concerns, these mechanisms can provide users with greater control over their personal information. This adaptability ensures that users feel more comfortable sharing data within social networks integrated with sensors and mobile devices, ultimately leading to increased user engagement and trust. Additionally, in trust-aware social sensing frameworks, self-adaptive privacy mechanisms can help balance the trade-off between personalization and privacy.

Hongchen et al. [30] address privacy concerns in social sensing, emphasizing the need for trust-aware models to mitigate privacy threats while enhancing personalization. By continuously monitoring user interactions and feedback, these mechanisms can dynamically adjust privacy settings and data disclosure behaviors to build and maintain user trust. This adaptability ensures that users’ privacy preferences are respected while still allowing for personalized social sensing experiences, leading to improved user satisfaction and engagement. Self-adaptive privacy mechanisms could also be essential in Vehicular Ad Hoc Networks to address the dynamic nature of vehicular communication and varying levels of privacy concerns among users, since trust management in Vehicular Ad Hoc Networks to secure communications and prevent attacks from malicious vehicles has already been investigated by Waheeb et al. [31]. By incorporating adaptive privacy controls into trust management frameworks, VANETs can better protect users’ sensitive information while enabling efficient and secure communication. These mechanisms can dynamically adjust privacy settings based on contextual factors such as location, traffic conditions, and user preferences, ensuring that privacy requirements are met without compromising communication reliability or security.

Finally, self-adaptive privacy mechanisms could be critical for protecting sensitive data collected by IoT devices in real time. By continuously monitoring data streams and adapting privacy measures based on changing threats and user preferences, these mechanisms can ensure that sensitive information is adequately protected from unauthorized access or disclosure. This adaptability enables IoT applications to maintain data privacy and security while still providing valuable insights and functionality to users [11].

Therefore, in the realm of self-adaptive privacy within cloud computing environments, several challenges and avenues for future research emerge. Although differential privacy algorithms show promise in safeguarding sensitive data, they often overlook users’ social profiles and preferences. Future research could focus on developing algorithms that better incorporate social context into privacy protection mechanisms. Furthermore, systems that adjust their operations based on privacy policies sometimes neglect critical factors like data collection limitations and minimization. Research efforts should strive to enhance software systems’ adaptiveness by addressing these gaps and ensuring compliance with evolving privacy regulations. While user-defined privacy preferences are vital, current approaches may disregard users’ contextual nuances and technical obstacles to managing their information effectively.

Future research might explore more user-friendly interfaces and decision-support systems, considering both user preferences and contextual factors. Providing users with greater control over their privacy settings is essential for enhancing transparency, trust, and user satisfaction. Future research might explore novel techniques for educating users about privacy risks and providing them with intuitive tools for managing their privacy preferences effectively. Therefore, crafting a comprehensive framework under the Cloud-InSPiRe project that considers social, software, and infrastructure factors is crucial for effective privacy protection in cloud computing environments. Collaboration between academia, industry, and regulatory bodies is essential to address emerging challenges and ensure that self-adaptive privacy practices evolve in tandem with technological advancements and users’ needs.

## Figures and Tables

**Table 1:** Groups’ needs for privacy protection.

Groups	Disclosure Practices (Information Shared)	Media and Services (Instagram, Messenger, Facebook)
Professional	About my job	Messenger: $\chi^{2}\left(1\right)$ = 7.917, *p* = 0.005, $\phi_{c}$ = 0.169
	Religious views	Messenger: $\chi^{2}\left(1\right)$ = 5.553, *p* = 0.018, $\phi_{c}$ = −0.142
	A short cv in my profile	Instagram: $\chi^{2}\left(1\right)$ = 5.470, *p* = 0.019, $\phi_{c}$ = −0.141
	I tag others in the photos I share	Instagram: $\chi^{2}\left(1\right)$ = 5.549, *p* = 0.018, $\phi_{c}$ = −0.142
Trade union	Photos of myself	Instagram: $\chi^{2}\left(1\right)$ = 4.502, *p* = 0.034, $\phi_{c}$ = −0.128
	About my hobbies	Facebook: $\chi^{2}\left(1\right)$ = 6.686, *p* = 0.010, $\phi_{c}$ = −0.156
		Instagram: $\chi^{2}\left(1\right)$ = 5.633, *p* = 0.018, $\phi_{c}$ = −0.143
	my location	Instagram: $\chi^{2}\left(1\right)$ = 7.107, *p* = 0.008, $\phi_{c}$ = −0.160
	I tag others in the photos I share	Instagram: $\chi^{2}\left(1\right)$ = 8.209, *p* = 0.004, $\phi_{c}$ = −0.172
Voluntary	Photos of myself	Instagram: $\chi^{2}\left(1\right)$ = 4.410, *p* = 0.036, $\phi_{c}$ = −0.126
		**WhatsApp:** $\chi^{2}\left(1\right)$ = 4.226, *p* = 0.040, $\phi_{c}$ = −0.124
Cultural	About my family	Messenger: $\chi^{2}\left(1\right)$ = 4.405, *p* = 0.036, $\phi_{c}$ = 0.126
	About my sexuality	Messenger: $\chi^{2}\left(1\right)$ = 11.908, *p* = 0.001, $\phi_{c}$ = 0.208
	Religious views	Messenger: $\chi^{2}\left(1\right)$ = 9.344, *p* = 0.002, $\phi_{c}$ = 0.184
	About my political views	Messenger: $\chi^{2}\left(1\right)$ = 8.041, *p* = 0.005, $\phi_{c}$ = 0.171
	My location	Messenger: $\chi^{2}\left(1\right)$ = 8.671, *p* = 0.003, $\phi_{c}$ = 0.177
	Contact information	Instagram: $\chi^{2}\left(1\right)$ = 3.863, *p* = 0.049, $\phi_{c}$ = −0.118
		**Messenger:** $\chi^{2}\left(1\right)$ = 3.888, *p* = 0.049, $\phi_{c}$ = 0.119
Scientific	About my job	Facebook: $\chi^{2}\left(1\right)$ = 9.700, *p* = 0.002, $\phi_{c}$ = 0.187
	About my hobbies	Instagram: $\chi^{2}\left(1\right)$ = 4.189, *p* = 0.041, $\phi_{c}$ = −0.123
	About my daily activities	Messenger: $\chi^{2}\left(1\right)$ = 4.597, *p* = 0.032, $\phi_{c}$ = −0.129
Environmental	Personal information	Messenger: $\chi^{2}\left(1\right)$ = 4.182, *p* = 0.041, $\phi_{c}$ = −0.123
Technological Interest	Photos of myself	Instagram: $\chi^{2}\left(1\right)$ = 8.102, *p* = 0.004, $\phi_{c}$ = −0.171
	About my hobbies	Instagram: $\chi^{2}\left(1\right)$ = 4.825, *p* = 0.028, $\phi_{c}$ = −0.132
	About my daily activities	Instagram: $\chi^{2}\left(1\right)$ = 5.751, *p* = 0.016, $\phi_{c}$ = −0.144

**Table 2:** Users belonging in both companionship and professional groups.

	Companionship and professional	Companionship	Professional
I share personal information	WhatsApp/N(144) $\chi^{2}\left(1\right)$ = 5.962, *p* = 0.015, $\phi_{c}$ = −0.203	Messenger/N(276) $\chi^{2}\left(1\right)$ = 6.844, *p* = 0.009, $\phi_{c}$ = 0.157	
I share photos of myself	WhatsApp/N(144) $\chi^{2}\left(1\right)$ = 12.038, *p* = 0.001, $\phi_{c}$ = −0.289	Instagram/N(276) $\chi^{2}\left(1\right)$ = 11.024, *p* = 0.001, $\phi_{c}$ = 0.200 Messenger/N(276) $\chi^{2}\left(1\right)$ = 6.517, *p* = 0.011, $\phi_{c}$ = 0.154	
I share information about my friends	Facebook/N(144) $\chi^{2}\left(1\right)$ = 4.237, *p* = 0.040, $\phi_{c}$ = 0.172	Messenger/N(276) $\chi^{2}\left(1\right)$ = 3.957, *p* = 0.047, $\phi_{c}$ = 0.120	
I share information about my job		Messenger/N(276) $\chi^{2}\left(1\right)$ = 5.227, *p* = 0.022, $\phi_{c}$ = 0.138	Messenger/N(276) $\chi^{2}\left(1\right)$ = 7.917, *p* = 0.005, $\phi_{c}$ = 0.169
I share information about my hobbies	WhatsApp/N(144) $\chi^{2}\left(1\right)$ = 8.456, *p* = 0.004, $\phi_{c}$ = −0.242	Instagram/N(276) $\chi^{2}\left(1\right)$ = 10.663, *p* = 0.001, $\phi_{c}$ = 0.197 Messenger/N(276) $\chi^{2}\left(1\right)$ = 5.632, *p* = 0.018, $\phi_{c}$ = 0.143	
I share information about my daily activities	Messenger/N(144) $\chi^{2}\left(1\right)$ = 4.782, *p* = 0.029, $\phi_{c}$ = −0.182 WhatsApp/N(144)$\chi^{2}\left(1\right)$ = 9.735, *p* = 0.002, $\phi_{c}$ = −0.260	Instagram/N(276) $\chi^{2}\left(1\right)$ = 10.115, *p* = 0.001, $\phi_{c}$ = 0.191 Messenger/N(276) $\chi^{2}\left(1\right)$ = 6.479, *p* = 0.011, $\phi_{c}$ = 0.153	
I share information regarding my sexuality	Messenger/N(144) $\chi^{2}\left(1\right)$ = 5.839, *p* = 0.016, $\phi_{c}$ = −0.201		
I share religious views	Messenger/N(144) $\chi^{2}\left(1\right)$ = 6.946, *p* = 0.008, $\phi_{c}$ = −0.220		Messenger/N(276) $\chi^{2}\left(1\right)$ = 5.553, *p* = 0.018, $\phi_{c}$ = −0.142
I share information about my political views		Instagram/N(276) $\chi^{2}\left(1\right)$ = 4.082, *p* = 0.043, $\phi_{c}$ = 0.122	
I state my location	Messenger/N(144) $\chi^{2}\left(1\right)$ = 5.143, *p* = 0.023, $\phi_{c}$ = −0.189		
I include contact information	Messenger/N(144) $\chi^{2}\left(1\right)$ = 8.663, *p* = 0.003, $\phi_{c}$ = −0.245		
I have included a short cv in my profile			Instagram/N(276) $\chi^{2}\left(1\right)$ = 5.470, *p* = 0.019, $\phi_{c}$ = −0.141
I tag others in the photos I share		Instagram/N(276) $\chi^{2}\left(1\right)$ = 5.520, *p* = 0.019, $\phi_{c}$ = 0.141	Instagram/N(276) $\chi^{2}\left(1\right)$ = 5.549, *p* = 0.018, $\phi_{c}$ = −0.142

**Table 3:** Relationship between group participation and participants’ self-protection strategies for companionship, leisure, scientific, environmental and technological interest groups.

Strategies	Companionship	Leisure	Scientific	Environmental	Technological Interest
Have changed initial privacy settings		N(273) $\chi^{2}\left(1\right)$ = 5.876 *p* = 0.015 $\phi_{c}$ = 0.147	N(273) $\chi^{2}\left(1\right)$ = 4.947 *p* = 0.026 $\phi_{c}$ = −0.135		
Often adjust privacy settings	N(272) $\chi^{2}\left(1\right)$ = 6.498 *p* = 0.011 $\phi_{c}$ = −0.155	N(272) $\chi^{2}\left(1\right)$ = 4.881 *p* = 0.027 $\phi_{c}$ = 0.134			N(272) $\chi^{2}\left(1\right)$ = 4.212 *p* = 0.040 $\phi_{c}$ = 0.124
Use a limited profile option			N(273) $\chi^{2}\left(1\right)$ = 5.420 *p* = 0.020 $\phi_{c}$ = −0.141	N(273) $\chi^{2}\left(1\right)$ = 3.865 *p* = 0.049 $\phi_{c}$ = 0.119	
Have excluded contact information from my profile			N(273) $\chi^{2}\left(1\right)$ = 5.200 *p* = 0.023 $\phi_{c}$ = −0.138		
Carefully consider the context (where am I) when I provide information		N(273) $\chi^{2}\left(1\right)$ = 5.940 *p* = 0.015 $\phi_{c}$ = 0.148			

**Table 4:** Relationship between group participation and participants’ self-protection strategies for professional, voluntary, religious and gender equality groups.

Strategies	Professional	Voluntary	Religious	Gender Equality
Have let privacy settings at default		N(273) $\chi^{2}\left(1\right)$ = 4.166 *p* = 0.041 $\phi_{c}$ = 0.124		
Do not accept friendship requests from strangers				N(273) $\chi^{2}\left(1\right)$ = 9.079 *p* = 0.003 $\phi_{c}$ = −0.182
Do not restrict access to the content I upload	N(273) $\chi^{2}\left(1\right)$ = 4.833 *p* = 0.028 $\phi_{c}$ = −0.133			
Untag myself from others’ photos		N(273) $\chi^{2}\left(1\right)$ = 6.121 *p* = 0.013 $\phi_{c}$ = −0.150		N(273) $\chi^{2}\left(1\right)$ = 3.921 *p* = 0.048 $\phi_{c}$ = 0.120
Familiar with the mechanisms provided by the platform to protect myself			N(273) $\chi^{2}\left(1\right)$ = 4.732 *p* = 0.030 $\phi_{c}$ = 0.132	

**Table 5:** Companionship and professional group’s self-protection strategies.

Self-Protection Strategy	Companionship and Professional	Companionship	Professional
During registration I did not give my real name	N(142) $\chi^{2}\left(1\right)$ = 6.208, *p* = 0.013, $\phi_{c}$ = −0.209		
I often adjust privacy settings		N(272) $\chi^{2}\left(1\right)$ = 6.498, *p* = 0.011, $\phi_{c}$ = −0.155	
I do not restrict access to the content I upload	N(142) $\chi^{2}\left(1\right)$ = 4.070, *p* = 0.044, $\phi_{c}$ = −0.169		N(273) $\chi^{2}\left(1\right)$ = 4.833, *p* = 0.028, $\phi_{c}$ = −0.133
I untag myself from others’ photos	N(142) $\chi^{2}\left(1\right)$ = 8.047, *p* = 0.005, $\phi_{c}$ = 0.238		
I have enabled the review of the posts in my profile	N(142) $\chi^{2}\left(1\right)$ = 3.945, *p* = 0.047, $\phi_{c}$ = 0.167		

**Table 6:** Companionship and voluntary group’s self-protection strategies.

Self-Protection Strategy	Companionship and Voluntary	Companionship	Voluntary
I have let privacy settings at default	N(142) $\chi^{2}\left(1\right)$ = 3.978, *p* = 0.046, $\phi_{c}$ = 0.167		N(273) $\chi^{2}\left(1\right)$ = 4.166, *p* = 0.041, $\phi_{c}$ = 0.124
I often adjust privacy settings		N(272) $\chi^{2}\left(1\right)$ = 6.498, *p* = 0.011, $\phi_{c}$ = −0.155	
I do not disclose personal information	N(142) $\chi^{2}\left(1\right)$ = 4.107, *p* = 0.043, $\phi_{c}$ = −0.170		
I do not disclose information about my family	N(142) $\chi^{2}\left(1\right)$ = 7.383, *p* = 0.007, $\phi_{c}$ = −0.228		
I untag myself from others’ photos			N(273) $\chi^{2}\left(1\right)$ = 6.121, *p* = 0.013, $\phi_{c}$ = −0.150
I control the information I upload	N(142) $\chi^{2}\left(1\right)$ = 3.842, *p* = 0.050, $\phi_{c}$ = −0.164		

**Table 7:** Social requirements.

SR: Social Requirement	
SR-1	Platforms should provide robust privacy settings, allowing users to manage the visibility of their views among different groups.
SR-2	Users should have the option to include or exclude a short curriculum vitae (CV) in their profile, with clear controls over its visibility to different audiences.
SR-3	Platforms should offer users granular/robust control over photo-tagging permissions to manage the visibility of tagged photos, to protect their privacy and that of others.
SR-4	Providing users with granular control over the visibility of hobby-related or personal interest information on their profiles.
SR-5	Offer effective location privacy settings and raise awareness among users about the risks associated with sharing their whereabouts.
SR-6	Respecting users’ privacy preferences regarding the disclosure of personal data or contact details.
SR-7	Provide protection mechanisms concerning the disclosure of routine behaviors or daily activity updates within communities on messaging platforms.

**Table 8:** Social protection measures.

SP: Social Protection Measure	
SP-1	Adjust privacy settings to maintain control over shared content.
SP-2	Restricting access to the uploaded content to ensure privacy boundaries.
SP-3	Untag themselves from others’ photos to manage their privacy.
SP-4	Change initial privacy settings from their profiles to mitigate privacy risks.
SP-5	Use a limited profile option to control their visibility.
SP-6	Exclude contact information from their profiles to mitigate privacy risks.
SP-7	Reject friendship requests from strangers to mitigate privacy risks.
SP-8	Pseudonymous or anonymous registration to accommodate users’ preferences for privacy.
SP-9	Privacy settings to allow users who participate in both groups to have greater control over who can access the content they upload by implementing robust access control mechanisms.
SP-10	Make informed decisions regarding personal information disclosure, including information about their families.

**Table 9:** Final selection of social requirements.

SR: Social Requirements	
SR-1	Platforms should provide robust privacy settings, allowing users to manage the visibility of their views or shared content among different groups.
SR-2	Users should have the option to include or exclude a short curriculum vitae (CV) in their profile.
SR-3	Platforms should offer users granular/robust control over photo-tagging permissions to manage the visibility of tagged photos.
SR-4	Providing users with granular control over the visibility of hobby-related or personal interest information on their profiles.
SR-5	Offer effective location privacy settings and raise awareness among users about the risks associated with sharing their whereabouts.
SR-6	Respecting users’ privacy preferences regarding the disclosure of personal data or contact details.
SR-7	Provide protection mechanisms concerning the disclosure of routine behaviors or daily activity updates.
SR-8	Change initial privacy settings from their profiles to mitigate privacy risks.
SR-9	Make informed decisions regarding personal information disclosure.
SR-10	Pseudonymous or anonymous registration to accommodate users’ preference for privacy.
SR-11	Reject friendship requests from strangers to mitigate privacy risks.

**Table 10:** Users’ groups and related social requirements.

Group	SR-1	SR-2	SR-3	SR-4	SR-5	SR-6	SR-7	SR-8	SR-9	SR-10	SR-11
Professional	X	X	X	X	X	X	X			X	
Companionship	X		X	X	X	X	X		X	X	
Trade union			X	X	X						
Voluntary			X						X		
Cultural						X					
Scientific				X		X	X	X		X	
Environmental							X				
Gender equality											X
Technological interest			X	X			X				

**Table 11:** Social, technical, and infrastructural requirements alignment.

	SR-1	SR-2	SR-3	SR-4	SR-5	SR-6	SR-7	SR-8	SR-9	SR-10	SR-11
TR-1 Compliance management						X			X		
TR-2 Transparency awareness						X		X	X	X	
TR-3 Context awareness	X	X	X	X	X		X		X		X
TR-4 User interaction	X		X	X	X		X		X		
TR-5 PII attribute management	X					X		X		X	
TR-6 Privacy control	X	X	X	X			X	X	X		X
TR-7 Intelligence management	X		X	X	X		X	X	X		X
IR-1 Storage management	X				X	X		X			
IR-2 Collaboration	X										X
IR-3 Developer guidance	X	X	X	X	X	X	X	X	X	X	X

## Data Availability

All research data have been anonymized. This study does not report any data.
